# Control of Hydroid Colony Form by Surface Heterogeneity

**DOI:** 10.1371/journal.pone.0156249

**Published:** 2016-06-03

**Authors:** Leo W. Buss, Evan D. Buss, Christopher P. Anderson, Michael Power, Joseph Zinter

**Affiliations:** 1 Department of Ecology and Evolutionary Biology, Yale University, New Haven, CT, United States of America; 2 Smithsonian Marine Station, Fort Pierce, FL, United States of America; 3 School of Engineering and Applied Science, Yale University, New Haven, CT, United States of America; 4 Center for Engineering Design and Innovation, Yale University, New Haven, CT, United States of America; UC Irvine, UNITED STATES

## Abstract

The colonial hydroid *Podocoryna carnea* grows adherent to surfaces progressing along them by a motile stolon tip. We here ask whether the stolon tip grows preferentially within grooves etched in silicon wafers. In a series of pilot experiments, we varied the dimensions of grooves and found that stolons did not utilize grooves with a width:depth of 5:5 μm or 10:10 μm, occasionally followed grooves 25:25 μm in size, and preferentially grew within grooves of a width:depth of 50:50 μm and 100:50 μm. We then grew colonies in grids, with fixed 50:50 μm width:depth channels intersecting at 90° every 950, 700, 450, or 150 μm. We find that stolons grew within grooves early in colony ontogeny, but remained restricted to them only in the grid pattern with channel intersections every 150 μm. Finally, we created a grid in the shape of the Yale Y logo, with channels of 50:50 μm width:depth and intersections every 100 μm. The resulting colonies conformed to that of the logo. Our findings demonstrate that stolons respond to surface heterogeneity and that surface etching can be used to fabricate microfluidic circuits comprised of hydroid perisarc.

## Introduction

Hydroids, like many colonial animals, often encrust surfaces in the sea [1–3]. They grow by the elongation and lateral branching of stolons. The resulting network of vascular canals can define the colony’s form. Form is typically species-specific; indeed, clone-specific in those species for which the topic has been studied [4–11]. The most detailed studies of clonal repeatability have been performed on colonies reared on glass surfaces in the laboratory. Under these conditions stolons tend to elongate without curvature and branch at set angles [4,5,8]. Whether this regularity is autogenous or reflects the homogeneity of the environment provided is an open question.

Two anecdotal observations motivate this study; both suggest that stolon tips of hydractiniid hydroids may be responsive to the microenvironment surrounding them. The first observation derives from long experience in growing *Hydractinia symbiolongicarpus* and *Podocoryna carnea* in the laboratory. We propagate these animals by tying a loop of thread around a microscope slide, slipping a tissue fragment from a stock colony under the thread, and removing the thread once the colony develops stolons that fix it to the slide. On very rare occasions removal of the thread reveals a stolon that has grown a substantial distance right along the margin of the thread without branching. This observation brings to mind another. Both of the hydroids mentioned grow as encrustations atop snail shells occupied by hermit crabs. Field studies of recruitment have led us to examine many young colonies, which had not yet covered their shells [12–14]. Not infrequently a shell is found bearing a colony of only a few polyps, but for which one or more stolons have traversed a substantial distance along a suture line on that shell.

These two observations made us wonder whether the behavior of a tip is an endogenous invariant, or whether tips may be responsive to aspects of the environment surrounding them. We have investigated this problem by growing colonies of the hydroid *Podocoryna carnea* on silicon wafers etched with a variety of groove patterns. We find that stolon tips prefer to grow in grooves indicating that tips have the capacity to detect and respond to their microenvironment.

## Methods

### Animals

**Fig 1:**
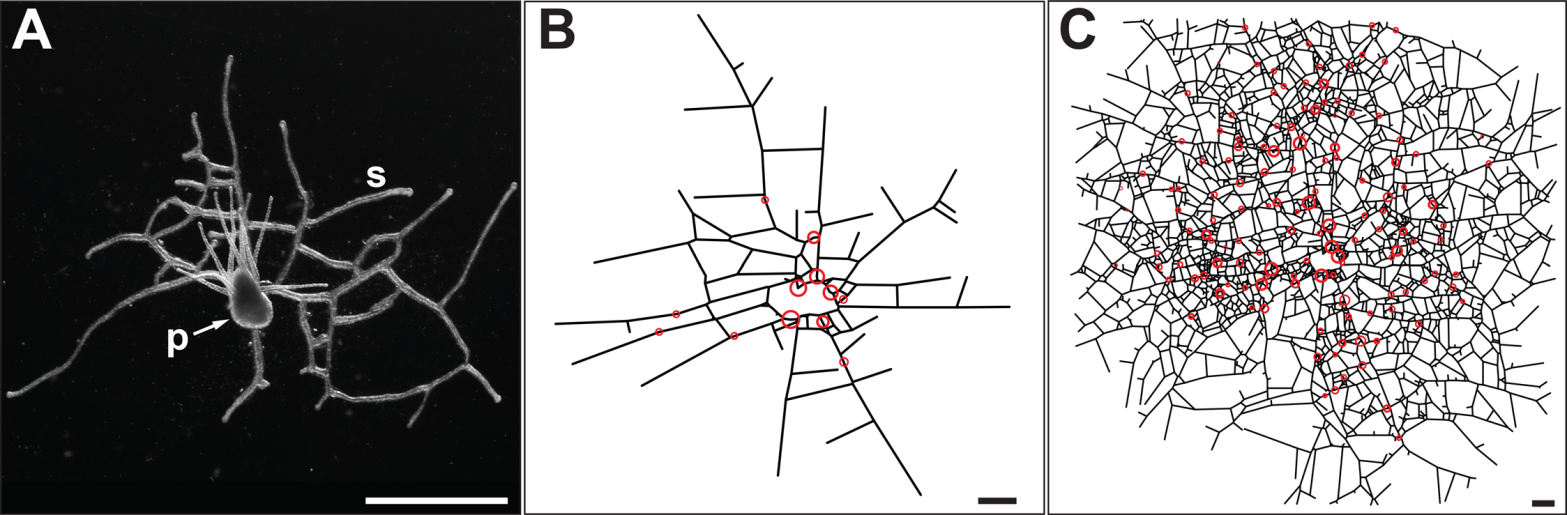
*Podocoryna carnea* (A) Top view of a young colony bearing a single polyp (p) and a ramifying hydrorhizal network of stolons (s). (B) Digitized image of a colony after 20 days growth. Circle denote locations of polyps, straight-line segments denote stolons. (C) Digitized image of the same colony after 58 days growth. Scale bar: 1 mm.

*Podocoryna carnea* is an athecate hydroid, which grows by elongation and lateral branching of stolons and the formation of new polyps atop existing stolons (Fig 1A). Young colonies are characterized by a stolonal network, called the hydrorhiza, of low connectivity (Fig 1A and 1B). Stolons fuse whenever lateral tips approach the flank of an existing stolon of the same colony. Hence the hydrorhiza of mature colonies becomes a densely connected network of stolons (Fig 1C). In such a state, further lateral branching is suppressed and medusa are formed, which embark on the sexual pelagic phase of the life cycle. Stolons are attached to the substratum [15,16] and hence the colony is effectively two-dimensional.

Our studies employ colonies of a single strain (P3) of *P*. *carnea*, collected from the intertidal of Lighthouse Point, New Haven CT in 1989 and propagated asexually in the laboratory ever since. The original collection was made under a permit issued by the Connecticut Department of Environmental Protection. *Podocoryna carnea* is neither a protected nor an endangered species.

Colonies were maintained under standard conditions [8]. Briefly, colonies were grown either on glass surfaces or silicon wafers. Clonal replicates were generated by explanting a small region of the hydrorhiza bearing 1–3 polyps and affixing them to the surface with a loop of quilting thread. After 2–7 days the colonies had attached and the thread was removed. Colonies were held in recirculating aquaria with daily exchanges of 25% of the seawater (31 ppt). Colonies were fed to repletion every other day with 3–4 day old *Artemia salina* nauplii.

### Etched Substrata

**Fig 2:**
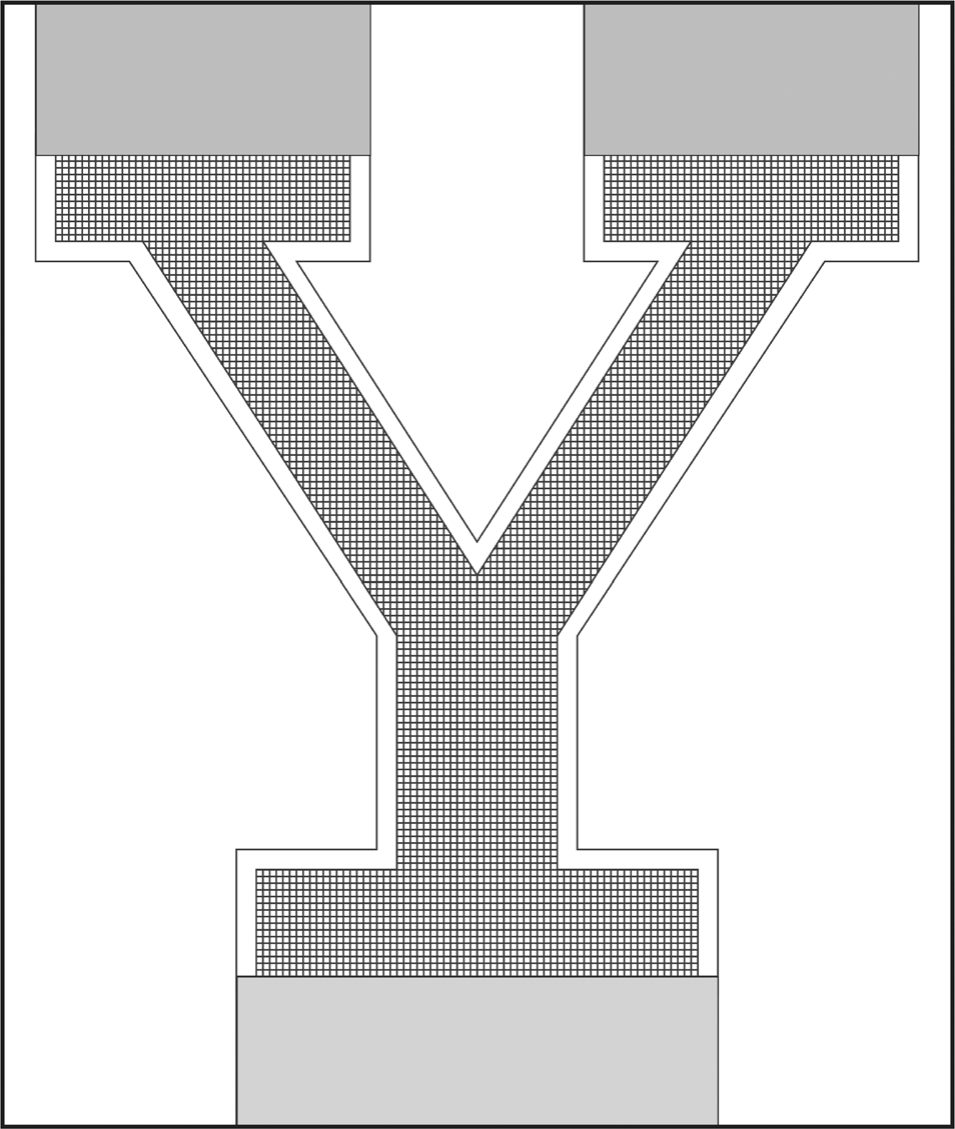
Yale Y logo. A grid pattern in the configuration of the Yale logo. Channels are 50 μm in width and 50 μm in depth, with intersections between channels occurring every 150 μm. The grey regions at the base and top of the arms of the logo are regions etched to a depth of 50 μm and used to initiate the growth of colonies from explants.

**Fig 3:**
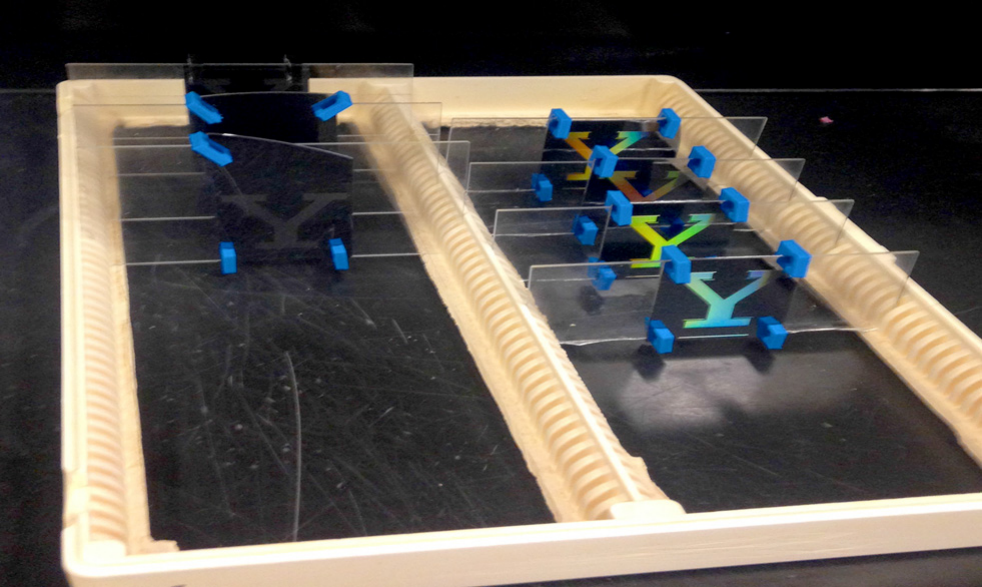
Silicon wafers etched with Yale logo, shown mounted on glass microscope slides in a plastic rack used to hold the wafers while in aquarium culture.

We reasoned that if colonies respond to surface micro-heterogeneities that it should be possible to induce a colony to grow in an arbitrary design. We chose the Yale Y logo as such a design. We performed two pilot experiments to establish the depth and spacing of grooves to utilize in the Yale Y experiment. In the first pilot experiment, a single silicon wafer was etched with grooves of varying width:depth (5:5, 10:10, 25:25, 50:50, 100:50 μm). An explant was placed adjacent to the grooves, and subsequent growth observed 14 days later. Digital images of colonies growing on etched surfaces were obtained using a Canon EOS 7D equipped with a Canon IS 100 mm macro lens. For comparison with stolons growing within grooves, we measured the width of 150 stolons growing on the same silicon wafers in regions of the wafer that was not etched. Stolon width was recorded as the distance from the one edge of the perisarc to the other on a line drawn perpendicular to the axis of growth. In a second pilot experiment, a series of grids were generated. All grids were etched with grooves at fixed width and depth of 50 μm, with the grooves intersecting at 90° angles every 950 (n = 4), 700 (N = 3), 450 (n = 4), or 150 (n = 6) μm. Colonies were imaged weekly for up to 13 weeks (S1 Table). Based on the results of the pilot experiments, 8 replicate silicon wafers were etched in the shape of the Yale University logo. Grooves were of a fixed width and depth of 50 μm with 90° intersections every 100 μm (Figs 2 and 3). Colonies were imaged monthly for 8 months (S1 Table).

When imaged from above, polyps obscure the hydrorhiza immediately underlying them. To visualize the hydrorhiza in these locations, we digested the living tissue from one replicate colony growing on a Yale Y silicon wafer and stained the perisarc using wheat germ agglutinin (WGA). To remove tissue, colonies were relaxed for 90 seconds in 2% urethane (Sigma) and placed in dH2O for 10 minutes in a Coplin jar. Colonies were then placed horizontally in Petri dishes and digested for 10–15 minutes in 10% KOH with intermittent gentle rinsing of the surface with the KOH solution by using a Pasteur pipette until tissue digestion was complete (ca. 10–15 min). Slides were then washed 3 x 5 minutes in dH2O. Chitin is a principal component of perisarc and WGA binds to the N-acetyl-D-glucosamine residues of chitin [17–20]. To stain with wheat germ agglutinin, slides were incubated for 20 minutes at room temperature in 25 μg/ml WGA-AlexaFluor 555 (Invitrogen) in PBS, washed 3x5 minutes in PBS, imaged in dH_2_O using a Texas Red filter set (Ex 560nm, Em 645nm) on a Zeiss Lumar fluorescence dissecting microscope.

Silicon wafers were etched using standard lithographic processes [21,22]. A photomask was created by a direct write laser (Heidelberg DWL66fs) on a 7 inch soda lime substrate using a.gdsii file generated in Adobe Illustrator. Subsequent lithography used a 150 mm silicon wafer coated with 1.5 μm of S1813 photoresist and pattern transfer using an EVG620 contact aligner. After development in MF319 developer, the wafer was etched in an Oxford 100 DRIE tool to the desired depth using a standard Bosch process [23]. Photoresist masking was removed by plasma ashing in an oxygen plasma. Wafers were then cleaned in a standard solvent cycle, rinsed in dH_2_0 and dried with nitrogen [24].

### Glass Surfaces

*Podocoryna carnea* was been the model organism for the study of colonial animal morphology [4,5,8,10,11,13,20,25–41]. We do not seek to replicate this considerable literature and will presume it understood that colonies do not naturally adopt a hydrorhizal network in the form of the Yale Y logo. We did however wish to compare stolon branching on glass substrata with the 90° branching imposed experimentally. To that end, we grew three colonies on glass microscope slides and imaged these colonies every other day for 58 days using a Zeiss Lumar dissecting microscope under the control of Zeiss Axiovision imaging software. We used these colonies to measure the angle at which lateral branches emerge from existing stolons (n = 50 branches/colony) for three 26 day old colonies. We manually digitized the image of one colony at 20 days and 58 days after explanting using a Wacom Cintiq 24D graphics monitor to generate the images used in Fig 1B and 1C.

We also measured the length of growing tips using colonies grown on glass cover slips (n = 21). Measurements were made at 200X using a Zeiss Axiovert compound microscope. Hydroid stolon tips are specialized tissues that undergo periodic extensions and retractions, with the former exceeding the latter in amplitude when the stolon is elongating [42–51]. Epithelial cells at the tip are attached to an acellular perisarc they secrete. A short distance proximal to the tip, the epithelium is no longer attached to the perisarc. We measured tip length as the distance between the tip to the distal-most point at which the epithelium was found detached from the perisarc. We measured the longer distance in those cases where the epithelia detached from the perisarc at differing distances from the tip on opposite sides of the stolon. Both the angle of branching and stolon length measurements was obtained from digital images using Zeiss Axiovision software.

The sample statistics for all experiments on both etched and glass surfaces are summarized in S1 Table.

## Results

### Grooves

**Fig 4:**
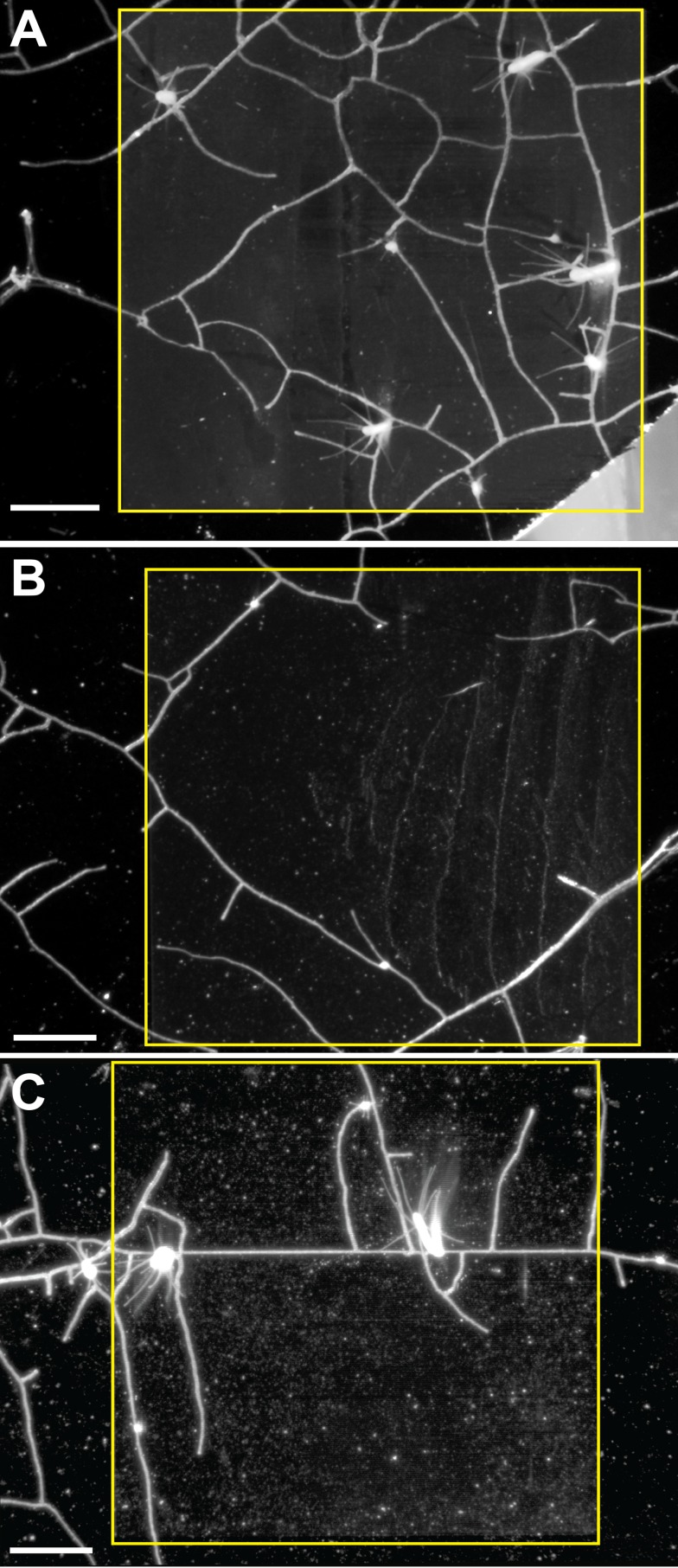
Shallow, narrow grooves. Groves run horizontally. Width:Depth (A) 5:5 μm, (B) 10:10 μm, (C) 25:25 μm. Regions of the wafer internal to the yellow bounding boxes were etched with grooves, regions outside the box were not etched. Scale bar = 1 mm.

**Fig 5:**
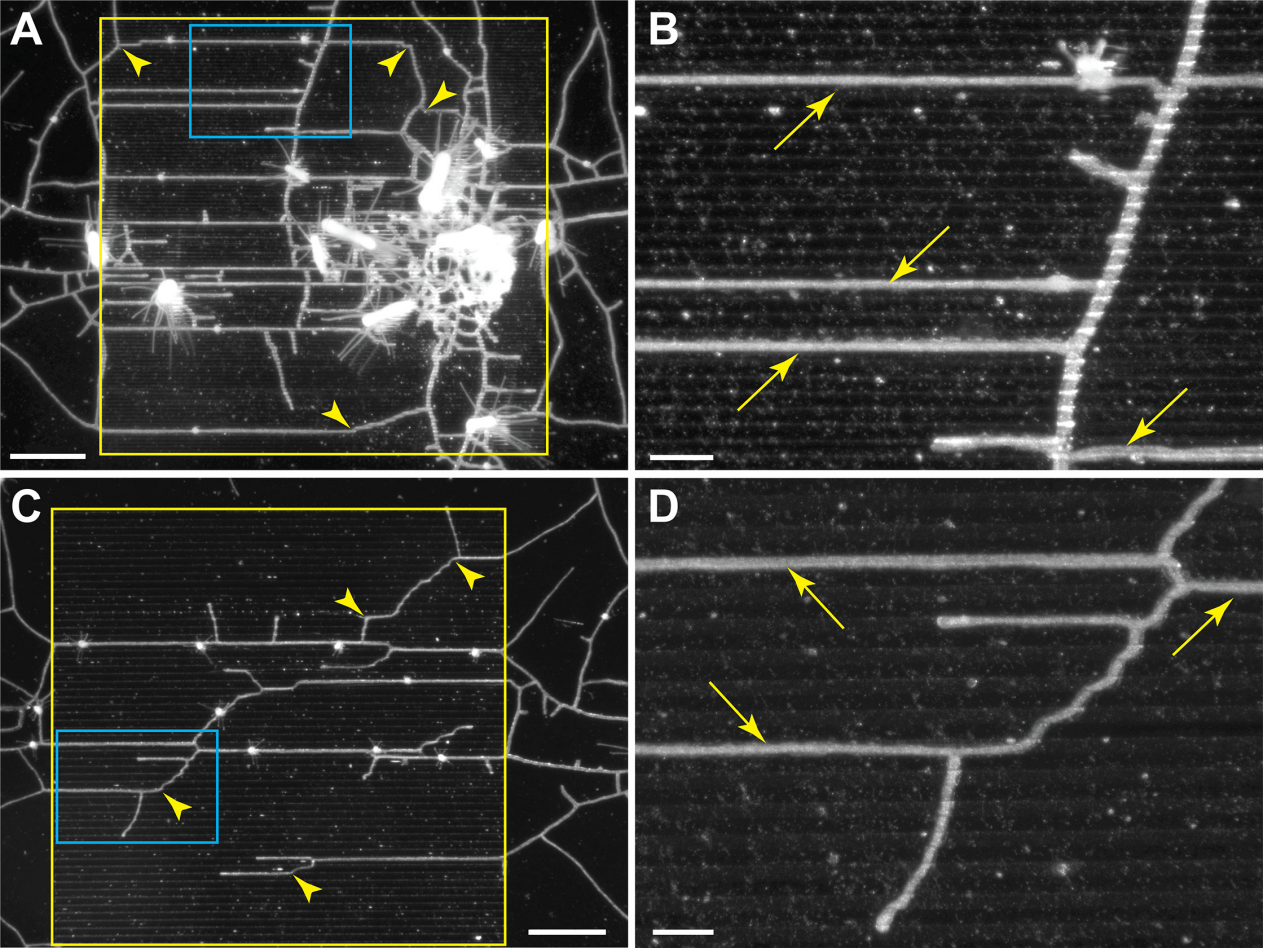
Wide, deep grooves. Groves run horizontally. Width:Depth (A, B) 50:50 μm, (C,D) 100:50 μm. Regions of the wafer internal to the solid yellow bounding boxes were etched with grooves, regions outside the box were not etched. Regions outlined in blue in A, C are shown at higher magnification in B, D. Scale bar for A, C = 1 mm, for B, D = 200 μm.

Stolons showed no propensity to grow within grooves with width:depth of 5:5 and 10:10 μm (Fig 4A and 4B). One stolon was found to grow within a groove of 25:25 μm width:depth, but lateral branches from that stolon did not (Fig 4C). Stolons were found predominantly within grooves of 50:50 and 100:50 μm width:depth (Fig 5A and 5C). In these cases, stolon tips often arose adjacent to a groove and elongated to form lateral branches within it (arrows in Fig 5B and 5D). Branches that crossed the grooved surface often located a groove and thereafter continued to grow within it (arrowheads in Fig 5A and 5C).

### Grids

**Fig 6:**
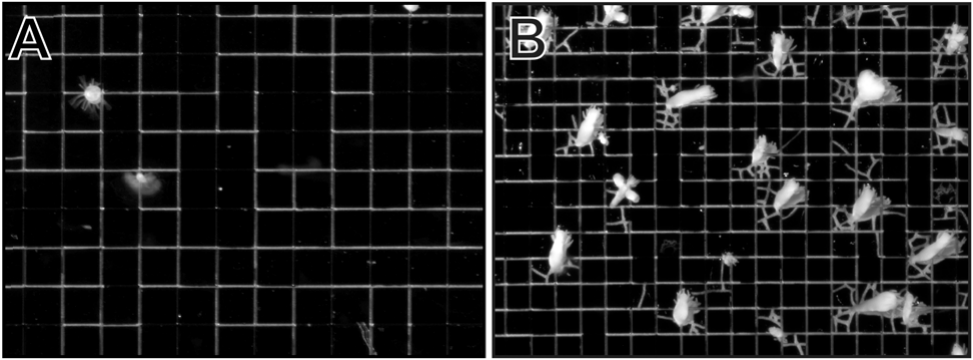
Grid with intersections between channels spaced at 700 μm. (A) After 45 days growth. (B) Same wafer after 57 days growth. Scale provided by grid pattern.

Colonies growing within grids with intersections spaced at intervals of 450, 700, and 900 μm (n = 4, 3, 4, respectively) all exhibited the same pattern of growth. In early time intervals, stolons were restricted to grooves, branching at intersections in the grid pattern with only rare exceptions (Fig 6A). As polyp buds and eventually mature polyps developed on these stolons, new stolons tips emerged from the base of these polyps and begin to populate the plateaus separating the grooves (Fig 6B). A 3 month time-series for a representative colony is shown in S1 Movie.

**Fig 7:**
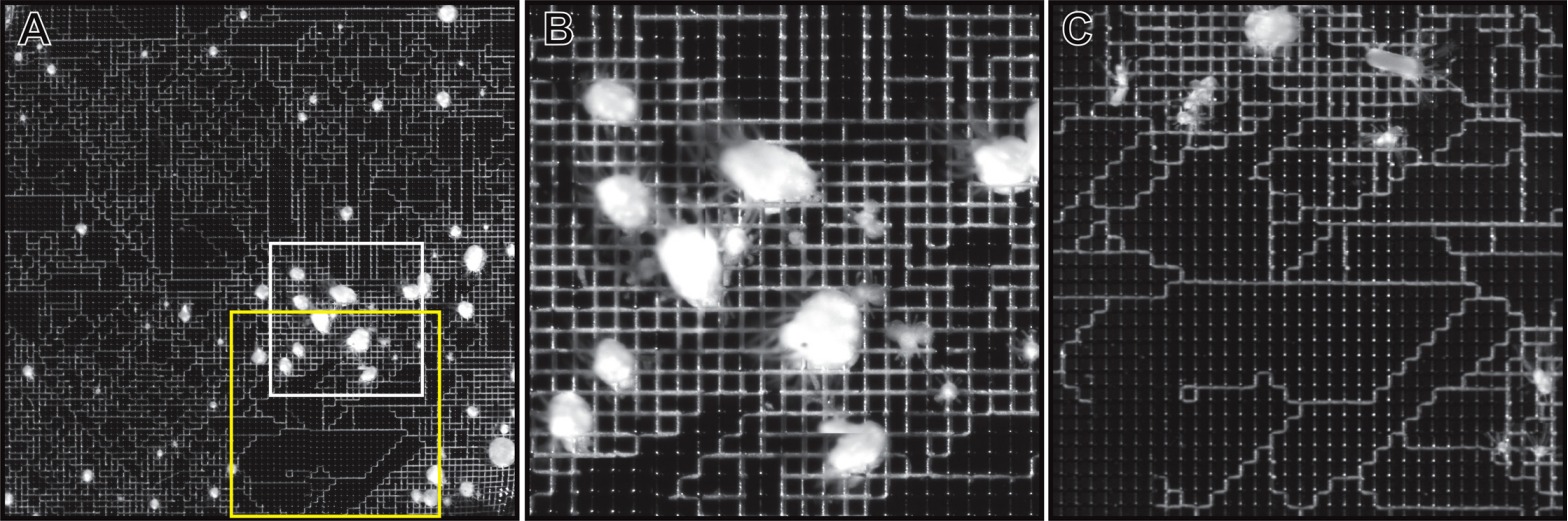
Grid with intersections between channels spaced at 150 μm. (A) After 45 days growth. (B) Magnification of region outlined in white in panel A. (C) Magnification of region outlined in yellow in panel A. Scale provided by grid pattern.

In contrast, colonies populating the 150 μm grid (n = 6) rarely ventured outside of grooves (Fig 7). Stolons of young colonies displayed a propensity to not only follow grooves, but also to grow within grooves in a zig-zag pattern (Fig 7C). A 3 month time-series for a representative colony is shown in S2 Movie.

### Yale Y

**Fig 8:**
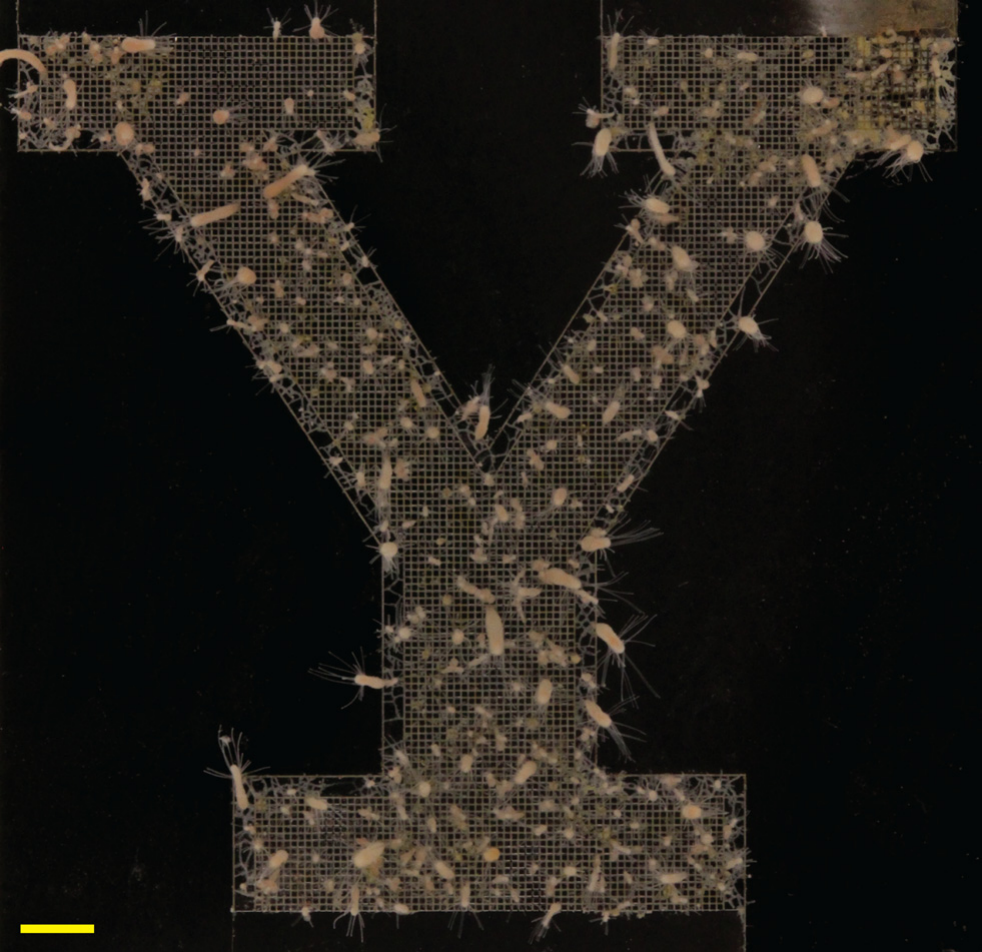
*Podocoryna carnea* adopting a colony form in the configuration of the Yale Y logo. Scale bar: 2 mm.

In all replicates, stolons grew within the grid pattern to generate a colony in the form of the Yale Y logo (n = 8). Stolons emerged from the grid when reaching the margins of the grooved region (Fig 8). A 6 month time-series for a representative colony is shown in S3 Movie.

**Fig 9:**
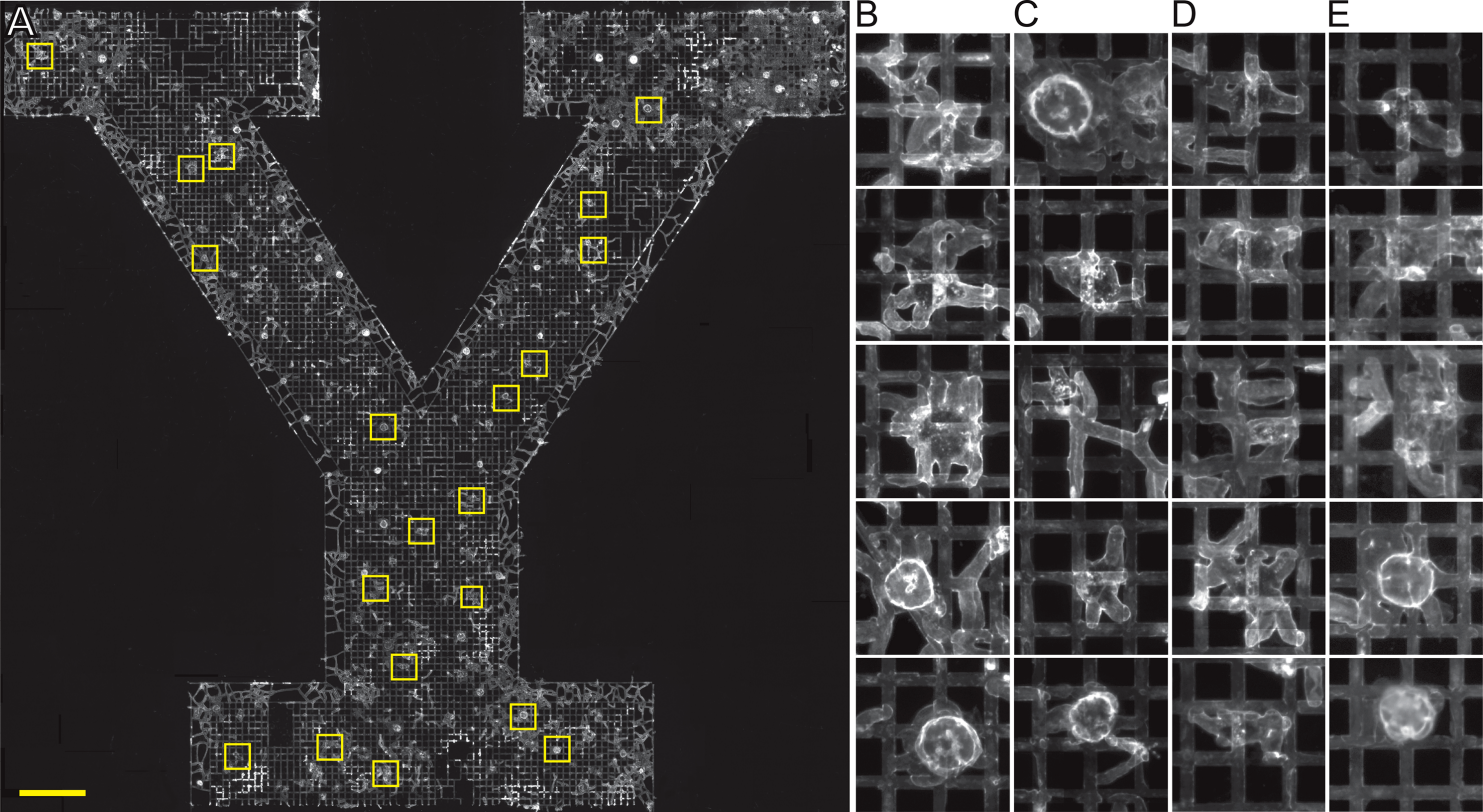
Hydrorhiza at the base of polyps. (A) Colony shown in Fig 8 with tissues removed and the perisarc stained with WGA. Regions outlined in yellow are location of selected polyps. Scale bar: 2 mm. (B-E) Hydrorhiza at the base of polyps in left hand (B), right hand (C) arms of the Y logo and in the shaft (D) and base (E) of the logo. Chloe, the opening in the perisarc from which a polyp emerges, outlined in yellow in (A) are shown running from top to bottom in B-D and from right to left in E. Scale for B-E provided by grid pattern (100 μm between channel intersections).

Polyps obscure view of the configuration of stolons beneath them (Figs 7 and 8). To determine whether stolons remained in the grid at the base of polyps, we removed the living tissue and stained the perisarc (Fig 9). Stolons were found to emanate from the polyp base even in the Yale Y configuration where stolons between polyps grew only within grooves.

### Stolon Morphometrics

**Fig 10:**
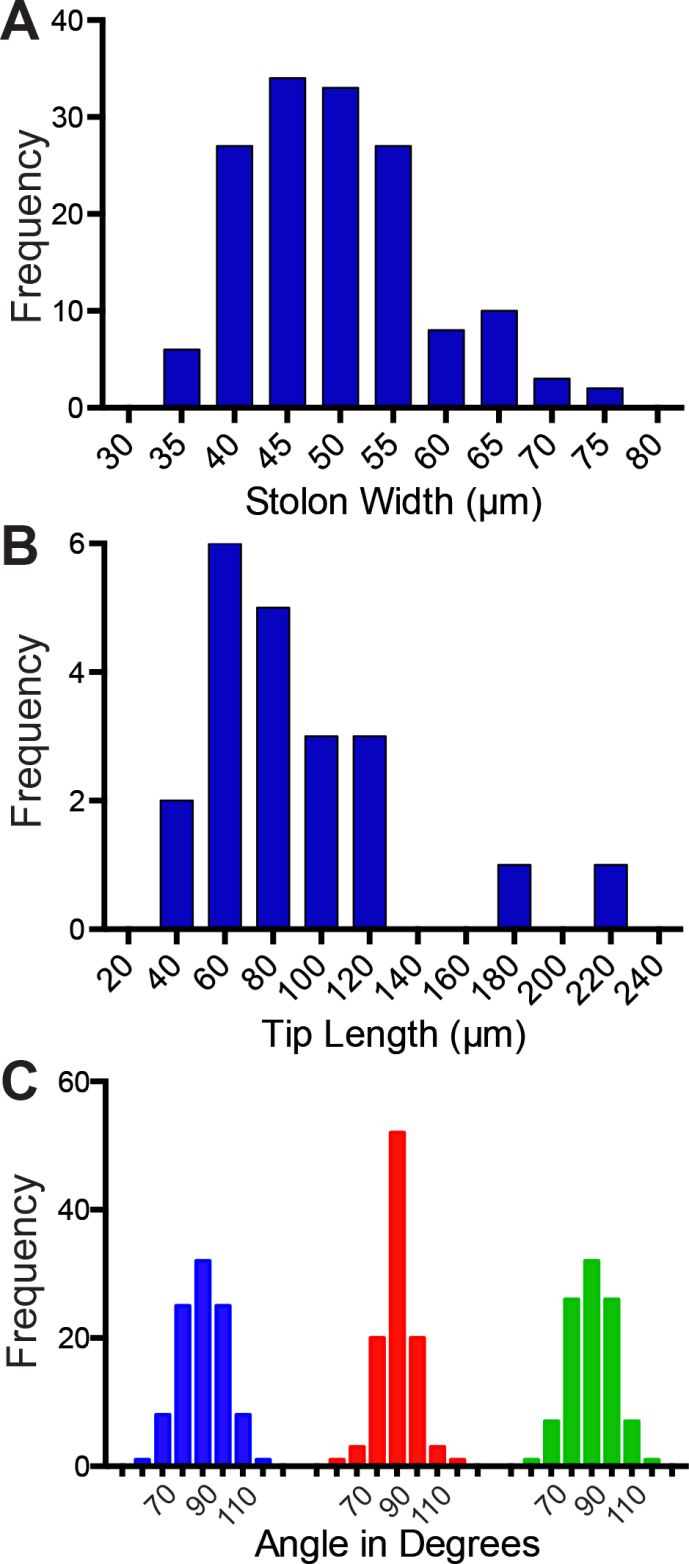
Morphometrics (A) Frequency distribution of stolon width (n = 150), measured perpendicular to the long axis of the stolon from perisarc to perisarc for colonies growing on silicon wafers. (B) Frequency distribution of stolon tip length (n = 21) measured from distal most point of tip to proximal point where the epithelia separates from the perisarc for colonies growing on glass. (C) Frequency distribution of angles subtended by 50 lateral branches for three 26 day old colonies growing on glass. Colors represent different colonies. Note each branch generates two angles which together sum to 180°.

The width of stolons, measured from perisarc to perisarc, was obtained for stolons growing on regions of the silicon wafers that had not been etched (Fig 10A). Note that the average width of stolons (49.5 +/- 8.5 μm) closely approximates the channel width preferred by stolons (Figs 5–8). The length of stolon tips was determined from observations of growing stolons at 200X (Fig 10B). Note that tip length frequently, but not always, exceeds the channel width in which stolons prefer to grow. Finally, the angles at which lateral branches emerge from stolons growing freely on glass closely approximated the 90° angle imposed on etched surfaces (Fig 10C).

### Raw Data and Specimens

The colonies growing in grids and in the form of the Yale logo have been accessioned to the Division of Invertebrate Zoology, Yale Peabody Museum (YPM#12228), where they are available for inspection. Time series of images of colonies growing in grids and the logo are available on the Dryad Data Repository (DOI:10.5061/dryad.ff893).

## Discussion

Our results show that stolon tips grow preferentially in grooves. We suspect that tips are capable of exhibiting this behavior because of an interplay that necessarily arises between the deposition of perisarc and elongation of the tip. Hydroid stolons literally grow inside of an acellular tube that they create [17,18,52]. The tip secretes perisarc, which is pliable when secreted and hardens with time [47,49,53–56]. We found that tips failed to follow grooves if the grooves were narrow and shallow (Fig 4). The stolon remained within the groove when the width of the groove approximated the perisarc-to-perisarc width of an unobstructed stolon (Figs 5 and 10A). We suggest that in a groove approximating the width of a stolon, the as yet unhardened perisarc conforms to the walls of the groove and thereby constrains the elongating stolon to extend within the groove.

At an intersection between two channels, the perisarc is not bounded by walls on either side for a distance of 50 μm. We found that in some cases the tip continued to extend within the groove leading to the intersection (Figs 5 and 6A). In other cases the tip turned regularly in a zig-zag fashion maintaining a constant angle of growth from its original branching point (Fig 7C). Only rarely did a tip make consecutive right (or left) turns to close onto itself (but see Fig 7C for two instances).

**Fig 11:**
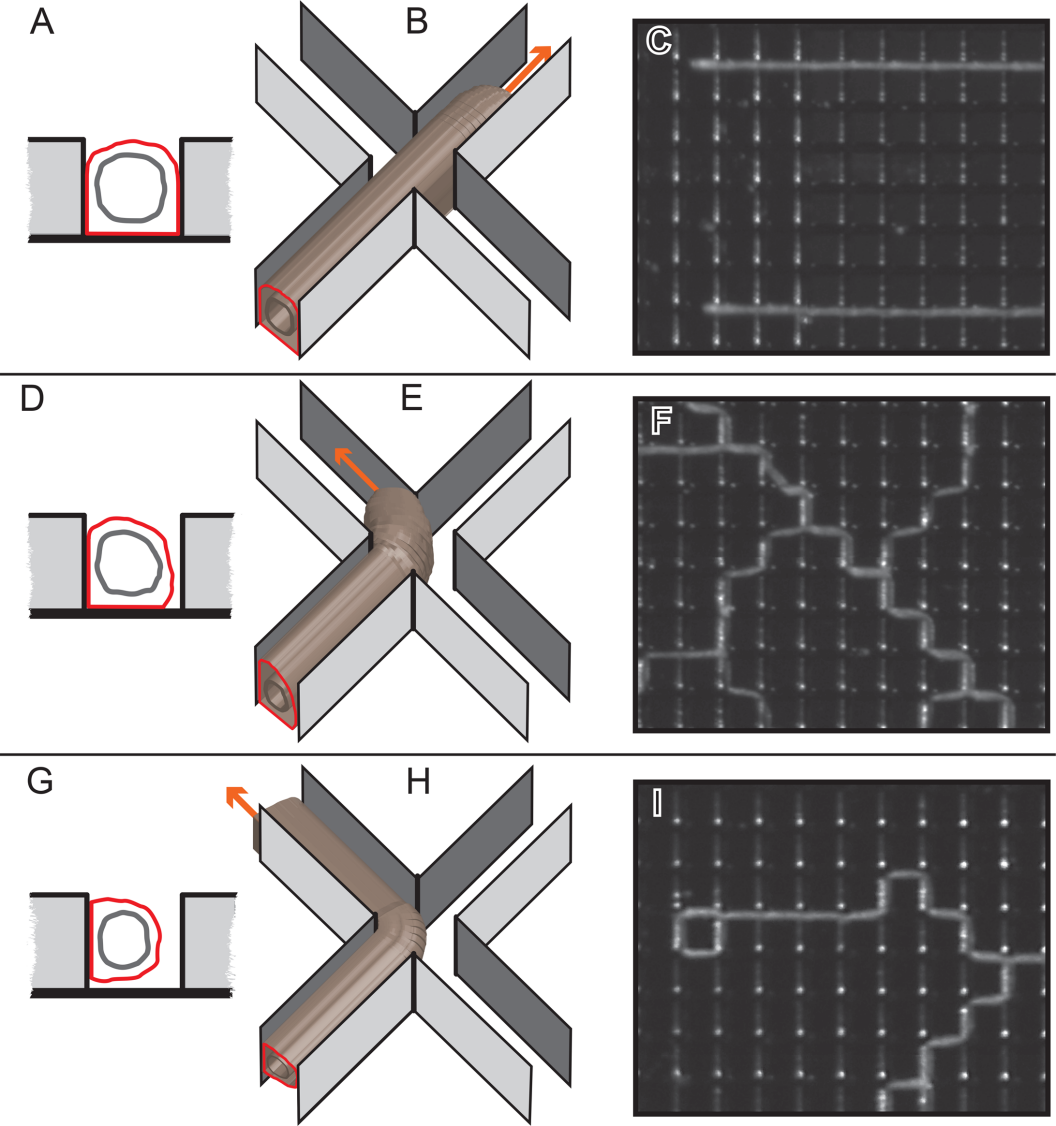
Hypothesized relationship between the positioning of the stolon within a channel and subsequent growth behavior. (A, D, G) A side view of a stolon attached to the base of the channel (A), the wall and the base (B), or the wall alone (C). Red indicates the boundary of the perisarc, grey indicates the boundary of the epithelia. (B, E, H) Top view showing the behavior of the tips hypothesized to result from the configurations shown in (A, D, G) respectively. Arrow indicates direction of growth. (C, F, I) Stolons growing within a grid with intersections every 150 μm, illustrating each the behaviors described.

We think that these behaviors may reflect the positioning of the stolon within the channel (Fig 11). We hypothesize that tips that are affixed to the base of the channel will continue to grow through intersections (Fig 11A–11C) without turning. Stolons that are affixed to the channel base and to one wall may, in passing into an intersection, first hug the wall to which it is attached bringing the tip into contact with the opposite wall, along which it thereafter extends (Fig 11D–11F), generating a zig-zag pattern of growth. Finally, if the stolon is affixed primarily to the wall of the channel, the tip may remain affixed to that wall generating a closed loop (Fig 11G–11I). Another variable that may be germane is the length of tip, which in some cases are longer than the intersections are wide, but can be shorter (Fig 10B). The hypotheses illustrated in Fig 11 can be readily tested by imaging the behavior of tips as they traverse intersections followed by sectioning of wafers to determine whether the hypothesized correlations with positioning of the stolons within channels hold. Alternatively, surfaces might be coated with materials antithetical to growth forcing stolons to grow only on walls or only on channels bases and observing whether the expected behaviors obtain.

The observation that large polyps in both grids (Fig 6B) and the Yale logo configuration (Fig 9) were found to give rise to stolons from their base is unsurprising. The polyp pumps nutrients from its gastric cavity into the hydrorhizal network [20,57] and an increase in the connectivity of stolons adjacent to polyps will reduce fluidic resistance.

The fact that stolon tips preferentially grow within grooves illustrates that the tip responds to surface micro-heterogeneity. One wonders if the capacity of tips to respond to their local environment is limited to selecting grooves over untextured surfaces. For example, predators or abrasions induced by the activities of their hermit crab hosts damage stolons and severed stolons subsequently generate new tips that reconnect to the colony. This process would be facilitated if the tip could preferentially grow on surfaces coated with chitin or some other perisarcal component. Other capacities are easily imagined; the tip may prefer to grow toward the shell’s aperture or away from potential competitors, either of which may potentially be detectable by water-borne cues. Hypotheses as to the capacities of stolonal tips to behave as sensory organs are readily testable. One need only grow a tip within a groove leading to an intersection where one path is provided with the material to be tested and the other not.

Our results may have a different utility. The hydrorhizal network of a colony exhibits an underlying statistical regularity, but the network configuration of two isogenic replicates is never identical [8,25,29,35]. This fact bedevils any investigation that seeks to standardize colony form. Growing colonies on etched surfaces should permit investigators to standardize hydrorhizal morphology during a broad swath of colony ontogeny.

Aside from potential utility to biological investigations, our findings may be of interest to engineers concerned with the design and fabrication of microfluidic devices. After removing the living tissues of a *Podocoryna carnea* colony, the chitinous perisarc remains (Fig 9). The result is a precise internal array of channels, a microfluidic circuit. Some hydroids, for example the sylasterine hydrocorals [58], secrete calcium carbonate and other invertebrates, silicon dioxides. Moreover, an increasing number of animals can be genetically transformed which may permit changes in the constituents and the material properties of these secretions and hence provide a new arena for fabrication technology.

## Supporting Information

S1 Movie Colony growing in grid pattern with channels of 50:50 width:depth and channel intersections every 700 μm. Movie encompasses 91 days of growth. Scale provided by the grid pattern.
(MOV)

S2 Movie Colony growing in grid pattern with channels of 50:50 width:depth and channel intersections every 150 μm. Movie encompasses 90 days of growth. Scale provided by the grid pattern.
(MOV)

S3 Movie Colony growing in the form of the Yale logo, with channels of 50:50 width:depth and channel intersections every 100 μm. Movie encompasses 179 days of growth. Scale provided by the grid pattern.
(MOV)

S1 Table Summary of experiments and sampling statistics. (DOCX)
