# Supplementary material for: Control of Hydroid Colony Form by Surface Heterogeneity
Source: PLoS One. 2016 Jun 3;11(6):e0156249. doi: 10.1371/journal.pone.0156249 (PMC4892489; doi:10.1371/journal.pone.0156249)
Supplement: S1 Table — (DOCX) [file pone.0156249.s004.docx]

**S1 Table**. Summary of experiments and sampling statistics

| Surface | Etching | Dimensions^1^ | Number of Replicates | # of Images  Acquired/  replicate | Sampling Interval^2^ |
| --- | --- | --- | --- | --- | --- |
| Silicon Wafer | Groove | 5:5 | 1 | 1 |  |
|  |  | 10:10 | 1 | 1 |  |
|  |  | 25:25 | 1 | 1 |  |
|  |  | 50:50 | 1 | 1 |  |
|  |  | 100:50 | 1 | 1 |  |
|  | Grid | 150 | 6 | 13 | 7 |
|  |  | 450 | 4 | 13 | 7 |
|  |  | 700 | 3 | 10 | 7 |
|  |  | 950 | 4 | 3 | 7 |
|  | Yale Y | 100 | 8 | 8 | 30 |
| Glass | None | n/a | 3 | 18 | 2 |

^1^ width:depth or grid separation with fixed 50:50 width:depth, in µm

^2^ days
